# Donor and Recipient Sex Matching and Corneal Graft Failure in High-Risk and Non-High-Risk Patients

**DOI:** 10.1155/2022/1520912

**Published:** 2022-04-16

**Authors:** Asaf Achiron, Tal Yahalomi, Idan Hecht, Nir Stanescu, Romi Achiron Noy, Zvia Burgansky-Eliash, Haggai Avizemer, Raimo Tuuminen, Oriel Spierer

**Affiliations:** ^1^Ophthalmology Division, Tel Aviv Sourasky Medical Center, Tel Aviv, Israel; ^2^Sackler Faculty of Medicine, Tel Aviv University, Tel Aviv, Israel; ^3^Department of Ophthalmology, Assuta University Medical Center, Ashdod, Israel; ^4^Department of Ophthalmology, Shamir Medical Center, Zerifin, Israel; ^5^Department of Ophthalmology, Meir Medical Center, Kfar Sava, Israel; ^6^Ophthalmology Department, Edith Wolfson Medical Center, Holon, Israel; ^7^Helsinki Retina Research Group, University of Helsinki, Helsinki, Finland; ^8^Department of Ophthalmology, Kymenlaakso Central Hospital, Kotka, Finland

## Abstract

**Purpose:**

It is controversial whether donor-recipient sex mismatch is a risk factor associated with corneal graft failure. The purpose of this study was to investigate the effect of sex mismatch on corneal graft failure in high-risk and non-high-risk patients.

**Design:**

A retrospective study.

**Methods:**

The medical charts of patients who underwent corneal transplantations by one surgeon between 2012 and 2017 were reviewed. Patients were defined as high-risk for failure if they had glaucoma, ocular surface disease, or corneal vascularization. Graft failure rates were compared using the Kaplan–Meier survival curves between sex matched and mismatched subjects and between male-to-female grafting and other patients.

**Results:**

One hundred and thirteen patients with a minimum follow-up of 18 months were included. In 62 non-high-risk patients, graft failure rates were similar between the sex mismatched and the sex matched recipients (*p*=0.645, log-rank) and in male donor to female recipient transplantations and in the other transplantations (*p*=0.496, log-rank). Analysis of fifty-one eyes of 51 high-risk graft recipients (mean age of 73.4 ± 12.7 years, *N* = 26 females) showed that graft failure rates were significantly higher in the sex mismatched than sex matched recipients (*p*=0.022, log-rank) and in male donor to female recipient transplantations than in the other transplantations (*p*=0.002, log-rank).

**Conclusions:**

Sex matching for every patient bares logistic difficulties; however, in patients who are at high-risk for graft failure, it may be a simple way to improve outcomes and better utilize corneal grafts.

## 1. Introduction

The cornea is an immune-privileged site due to various factors, including the absence of blood and lymphatic vessels, blood-eye barrier, and immunomodulatory factors. Thus, any cornea deemed appropriate for transplantation can be transplanted into any recipient, regardless of the recipient's age, sex, and HLA type [1]. However, corneal graft rejections and failures are common, reported to be 15–18% in full-thickness grafts and 4–9% in posterior lamellar grafts, during 10 years of follow-up [2]. Immunological inflammation may contribute some risk to the overall reported five-year graft survival of 71% (95% CI: 69–73%) [3].

Male-to-female (M⟶F) corneal grafts have been reported to have a lower graft survival rate compared to matched corneal transplants [4]. This finding has been suggested to result from mismatching in the H-Y (male) antigen. H-Y antigen is derived from a group of proteins encoded on the Y chromosome of male donors, which elicits an immune response in females but not in males. This antigen is known to be associated with the reduced long-term kidney and hematopoietic stem-cell graft survival rates seen in male-to-female transplantations [5–7]. Nevertheless, a literature review on sex mismatching and corneal graft outcomes demonstrated contradictory results [5, 8–13]. A large national cohort study conducted by the National Health Service (NHS) Blood and Transplant in the UK showed that sex mismatched corneas were at a greater risk for failure, regardless of diagnosis or surgery type [8]. However, two recent studies from the US reported that donor-recipient sex combinations did not affect rejection episodes or graft survival [11, 12].

In low-risk patients undergoing Descemet's stripping endothelial keratoplasty (DSEK) or Descemet membrane endothelial keratoplasty (DMEK), where the risk for failure is low, sex mismatching was not associated with graft survival [12]. A recent randomized controlled trial showed no significant advantage of sex matching compared to random graft assignment in a heterogeneous study population [14].

High-risk keratoplasty is associated with low success rates due to a high incidence of immune-mediated graft rejection [15] and may require systemic immunosuppression to prevent rejection [16]. Given the sparse data in the literature regarding the possible importance of sex matching and corneal transplantation in high-risk patients, we aimed to evaluate whether sex mismatching is associated with a lower survival rate of corneal grafts in high-risk patients as compared to non-high-risk patients.

## 2. Materials and Methods

A retrospective chart review of all patients of age ≥18 years who underwent primary corneal transplantation at the E. Wolfson Medical Center, Sackler Faculty of Medicine, Tel Aviv University, between 2012 and 2017 was carried out. DMEK was performed at our institute starting in 2017. The steep learning curve associated with this procedure may bias the results. Thus, DMEK cases done in 2017 were not reviewed.

**Figure 1:**
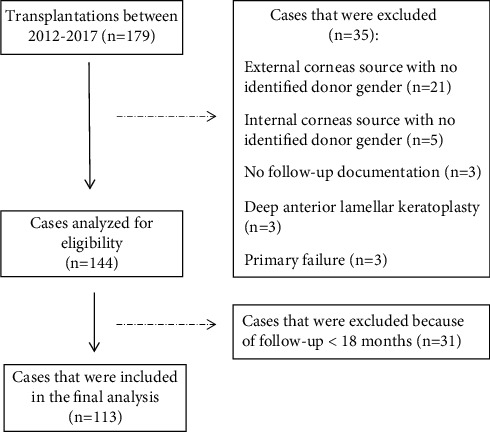
Flow diagram of the patients included in the analysis.

Primary graft failures (nonclearing grafts within 2 weeks of transplantation) were excluded as they represent complexities with donor tissues unrelated to donor-recipient matching [17]. Deep anterior lamellar keratoplasties were also excluded as the donor grafts did not contain endothelial cells, which might elicit an immunological response that is different from grafts that do contain endothelium [18]. Only patients with a follow-up of at least 18 months were included. High-risk factors for failure included the presence of glaucoma, ocular surface disease, and corneal vascularization [8].  Figure 1 shows the flow diagram of the patients included in the analysis.

A single surgeon (HA) performed all surgeries. Postoperative visits were scheduled for day 1; weeks 1, 2, and 4; and months 3 and 6, and then every 6 months. Topical antibiotics t.i.d. and corticosteroid drops (dexamethasone 0.1%) t.i.d. were applied after PK and DSEK. Antibiotics were stopped after one week and corticosteroid drops were tapered down, usually to b.i.d. after one month and once daily after an additional 3 months. After that, a less potent corticosteroid (fluorometholone 0.1%) was used once daily. In high-risk patients, usually a more potent corticosteroid (dexamethasone 0.1%) is used once daily.

Graft failure was defined according to the cornea preservation time study as the occurrence of a corneal graft that was initially clear and subsequently became cloudy, preventing recovery of useful vision and requiring additional corneal transplantation [19]. Failure was defined as corneal edema without signs of inflammation (keratic precipitates and Khodadoust line) and after a course of topical steroids.

Failure-free graft survival was defined as the period from keratoplasty to the postoperative meeting in which the patient was diagnosed with failure. The study was approved by the Institutional Review Board of the E. Wolfson Medical Center and complied with the principles outlined in the Declaration of Helsinki.

### 2.1. Statistical Analysis

The clinical parameter distribution for normality was analyzed by the Shapiro–Wilk test. For two-group comparisons, parametric variables were analyzed with the Student's *t*-test and nonparametric variables with the Mann–Whitney *U* test. Categorical data was analyzed with Pearson's chi-square test or Fisher's exact test when values in any of the cells of a contingency table were below five. The donor-recipient relationship was divided according to sex matched status. The sex matched group included male to male (M⟶M) and female to female (F⟶F) donations, while the sex mismatched group included male to female (M⟶F) and female to male (F⟶M) donations. For survival, the Kaplan-Meier analysis with log-rank (Mantel–Cox) was applied. IBM SPSS Statistics 27 (IBM Corp., Armonk, NY) was used for statistical analysis. Unless otherwise specified, values represent the mean ± standard deviation.

## 3. Results

One hundred and thirteen eyes of 113 patients with a mean follow-up time of 3.2 ± 1.3 years were included.

In the 62 non-high-risk patients, the mean donor and recipient ages were 63.0 ± 9.1 and 75.1 ± 9.7 years, respectively. Male-to-female sex distribution was 47 : 15 (76 : 24%) and 18 : 44 (29 : 71%), among donors and recipients, respectively. There were 23 (37.1%) cases of sex-matched transplantations. Over a mean follow-up time of 3.2 ± 1.3 years, 6 (9.7%) cases of graft failure were documented. No difference was observed in graft failure among the sex mismatched when compared to sex matched recipients (*p*=0.645). No difference was observed in graft failure among the male donor to female recipient transplantations when compared to other transplantations (*p*=0.496).

In the 51 high-risk recipients, the mean donor and recipient age were 57.5 ± 15.8 and 73.4 ± 12.7 years, respectively. Male-to-female sex distribution was 35 : 16 (69 : 31%) and 25 : 26 (49 : 51%) among donors and recipients, respectively.

DSEK was performed in 26 (51%) cases and penetrating keratoplasty (PK) in 25 (49%) cases. There were 33 (65%) cases of sex-matched transplantations. Over a mean follow-up time of 3.0 ± 1.4 years, 11 (21.6%) cases of graft failure were documented.

There were no differences in the baseline characteristics between the sex match and mismatch groups (Table 1). Baseline variables were also similar between the male-to-female corneal graft recipients and the other recipients (Table 1).

**Figure 2:**
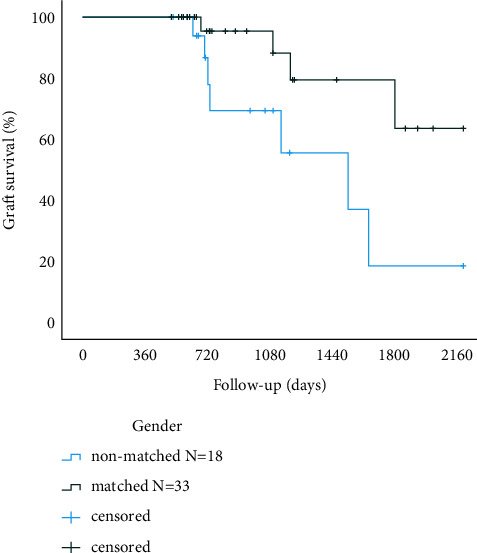
Corneal graft Kaplan–Meier survival curves in gender matched and mismatched high-risk patients (*p*=0.022, log-rank test).

**Figure 3:**
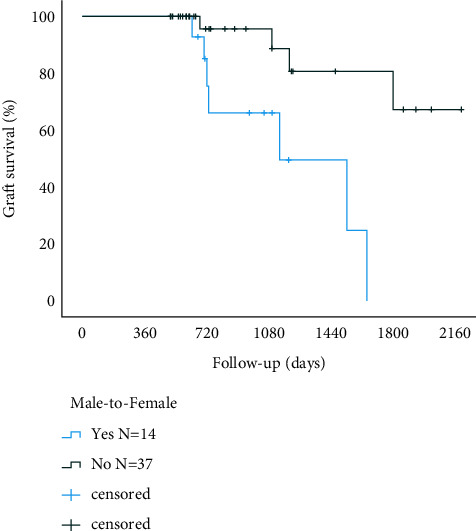
Corneal graft Kaplan–Meier survival curves in male-to-female recipients and other high-risk patients (*p*=0.002, log-rank test).

The graft failure rate was significantly higher among the sex-mismatched when compared to sex matched recipients (*p*=0.022; Figure 2). The graft failure rate was also more common among the male donor to female recipient transplantations when compared to other high-risk transplantations (*p*=0.002; Figure 3).

**Figure 4:**
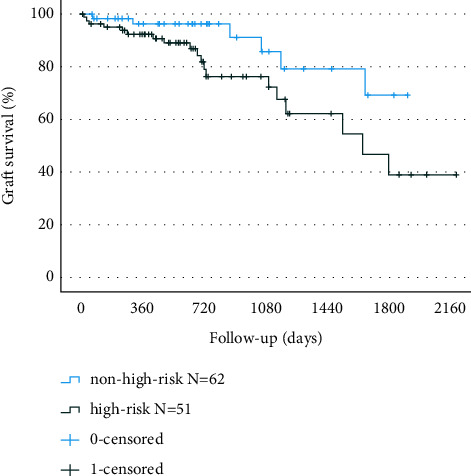
Corneal graft Kaplan-Meier survival curves in non-high-risk and high-risk patients (*p*=0.034, log-rank test).

A comparison of baseline characteristics between non-high-risk and high-risk patients is shown in Table 2. The graft failure rate was significantly higher among the high-risk patients as compared to the non-high-risk patients (*p*=0.034, Figure 4).

## 4. Discussion

In this study, we found that in high-risk patients having comorbidities of glaucoma, ocular surface disease, or corneal vascularization, sex mismatching was associated with an increased risk for graft failure after a mean of three years of follow-up. This was emphasized especially in the male-to-female corneal transplantations. Thus, according to our study, women with high-risk characteristics for corneal graft failure, facing corneal transplantation, could benefit from female donors.

Currently, corneal transplantation guidelines do not support corneal matching due to the extensive immune privilege of the cornea [20]. The immune-regulating mechanisms include an afferent blockade of the immune response to foreign antigen by both the absence of antigen-presenting cells and a lack of corneal vascularization that prevent donor antigen from reaching host lymph nodes [21]. In addition, Th1 is downregulated via the Fas ligand in an anterior chamber-associated immune deviation response which leads to an immune-privileged environment [22]. However, despite this mechanism, immunological rejections do occur, with a higher rate in PK (15–18%) than in anterior lamellar (0–6%) and posterior lamellar (DSEK: 4–9% and DMEK: 1.2%) keratoplasty [2, 11, 23–25]. Still, in keratoplasty, sex or HLA matching is not performed widely. This is probably due to logistic barriers and controversial evidence in the literature.

In 2006, Böhringer et al, [4] were the first to suggest that sex matching could improve prognosis in corneal transplantation. In their study of 2 years follow-up after PK patients, mismatched H-Y (male⟶female) corneal graft survival was lower compared to matched grafts (77% vs. 88%, *p*=0.02). Hopkinson et al. [8] reported in Fuchs endothelial dystrophy and keratoconus patients that in both PK and endothelial keratoplasty, sex mismatch, and the combination of male donor and female recipient, in particular, was at greater risk of failure. In contrast, a recent study by Kim et al. [10] showed that in low-risk patients, sex matching did not affect graft survival in cases of primary PK. Data from the Swedish Cornea Transplant Registry showed that in DSEK, when the donor was male, there was a significantly lower 2-year graft survival rate than when the donor was female [13]. A recent study by Price et al. comparing DSEK and DMEK showed that sex matching did not affect the 5-year graft survival in DSEK and DMEK [12]. An updated report from the cornea preservation time study on DSEK patients showed that donor-recipient sex mismatch was not related to rejection [11]. In a recent study, sex matching was not a predictive factor for rejection or graft failure nor seemed to influence the incidence of failures concerning the patient's risk category in one year of follow-up [26].

Regarding the effect of sex mismatching on corneal graft outcomes in high-risk patients, data is sparse. Hopkinson et al. have reported that in high-risk patients, sex mismatching results in a higher graft failure rate, although this is less pronounced than in low-risk conditions [8]. A recent study by Kim et al. showed that in high-risk patients, the time to failure was longer in H-Y matched subjects when compared to nonmatched donor-recipients [10].

Donor-recipient sex matching may be more significant on corneal graft outcomes in PK than in posterior lamellar keratoplasty. This could be explained in several ways. First, the thinner posterior lamellar graft has a lower antigen load; therefore, it is less immunogenic than the full-thickness graft [27]. Second, the posterior lamellar graft is located in the anterior chamber, not exposed to the outer surface, which includes antigen-presenting cells and stromal vessels [28]. Third, posterior lamellar grafts do not contain an epithelial layer that has different immunogenicity and sensitivity to rejection, independent of the stroma or endothelium [29]. The immunological mechanism behind the sex mismatching and increased risk for graft failure observation is speculated to be mediated by naive female-recipient lymphocytes (CD_4_^+^ T cells) that recognize H-Y minor histocompatibility antigens, which include intracellular proteins encoded by the Y chromosome of a male donor. Following H-Y antigen recognition, H-Y antibodies were observed to develop in mismatched transplantations in other organs (kidneys and hematopoietic cells) [30]. These antibodies are related to graft rejection and graft versus host disease [6]. In addition, a cytotoxicity response has also been linked to an H-Y male-specific minor histocompatibility antigen recognition [4]. The sex mismatching effect in high-risk patients in the current study, observed in a mean of 3 year of follow-up after corneal transplantation, may suggest a chronic slow inflammation, with continuing damage to the corneal graft until its failure.

Limitations of our study include its retrospective design and the lack of HLA genetic matching data. The H-Y antigen is not present in all males, but only in those with HLA-A1. The genetic frequency of the HLA-A1 allele is population dependent and is present in 20% of the general population in the UK, 18% of the general population in Australia, and only 15% of the Jewish population in Israel [31]. In addition, the study did not include DMEK patients. As this was a retrospective study, there were differences in the protocol of treatment between patients, and the impact of treatment diversity on graft survival could not be evaluated. The main study strength includes the consistency of outcomes assessment across procedures at a single center by a single surgeon.

## 5. Conclusions

In high-risk patients, donor matching, and predominantly female-to-female donor matching, may lead to a lower corneal graft failure rate. Sex matching for every patient bares logistic difficulties; however, it may be a simple way to improve outcomes and better utilize corneal grafts in high-risk patients. We suggest that in high-risk patients where corneal graft failure is more likely, sex matching could be suggested to improve outcomes.

## Figures and Tables

**Table 1:** Baseline characteristics of the 51 high-risk patients.

Parameter	Gender		*p*	Male-to-female		*p*
	Mismatch (*N* = 18)	Match (*N* = 33)		Yes (*N* = 14)	No (*N* = 37)	
Donor age	56.1 ± 11.9	58.1 ± 17.4	0.733	53.3 ± 11.1	58.7 ± 16.9	0.399
Recipient age	74.1 ± 10.4	73.0 ± 13.9	0.786	78.2 ± 5.1	71.9 ± 14.0	0.173
Follow-up (days)	1072.1 ± 496.9	1087.7 ± 510.8	0.940	1042.7 ± 297.5	1098.9 ± 559.9	0.758
Indication for keratoplasty						
Previous failed graft	7 (38.9)	15 (45.4)	0.651	7 (50.0)	15 (40.5)	0.543
PBK	5 (27.8)	13 (39.4)	0.407	5 (35.7)	13 (35.1)	0.969
FED	1 (5.6)	—	0.353	1 (7.1)	—	0.275
Corneal perforation	1 (5.6)	3 (9.1)	1.000	—	4 (10.8)	0.565
Corneal infection	1 (5.6)	2 (6.1)	1.000	—	3 (8.1)	0.552
Others	3 (16.7)	—		1 (7.1)	2 (5.4)	
Surgery type						
DSEK	11 (61.1)	15 (45.5)	0.285	10 (71.4)	16 (43.2)	0.116
PK	7 (38.9)	18 (54.5)	0.285	4 (28.6)	21 (56.8)	0.116
Graft size (mm)	8.2 ± 0.3	8.4 ± 0.2	0.134	8.2 ± 0.3	8.5 ± 0.2	0.091
High-risk factors for graft failure						
Glaucoma	14 (77.8)	22 (66.7)	0.405	11 (78.6)	25 (67.6)	0.513
Ocular surface disease	2 (11.1)	8 (24.2)	0.462	2 (14.3)	8 (21.6)	0.707
Corneal stromal vascularization	2 (11.1)	1 (3.0)	0.282	2 (14.3)	1 (2.7)	0.179

The data are given as the mean ± SD for continuous variables and absolute numbers with proportions for categorical variables. For two-group comparisons, parametric variables (age and follow-up) were analyzed with Student's *t*-test, while nonparametric variable (graft size) with the Mann–Whitney *U* test. The categorical data were analyzed with Pearson's chi-square test or Fisher's exact test when values in any of the cells of a contingency table were below five. PBK: pseudophakic bullous keratopathy; FED : Fuchs endothelial dystrophy; DSEK : Descemet stripping endothelial keratoplasty; PK: penetrating keratoplasty.

**Table 2:** Baseline characteristics comparison between non-high-risk and high-risk patients.

Parameter	Non-high-risk (N = 62)	High-risk (N = 51)	*p*
Age (years)			
Donor	63.0 ± 9.1	57.5 ± 15.8	0.084
Recipient	75.1 ± 9.7	73.4 ± 12.7	0.511
Gender (male : female)			
Donor	47 : 15 (76 : 24%)	35 : 16 (69 : 31%)	0.394
Recipient	18 : 44 (29 : 71%)	25 : 26 (49 : 51%)	0.029
Follow-up (days)	1163.8 ± 488.7	1084.0 ± 500.1	0.542

The data are given as the mean ± SD for continuous variables and absolute numbers with proportions for categorical variables. For two-group comparisons, parametric variables (age and follow-up) were analyzed with Student's *t*-test and categorical data (gender) with Pearson's chi-square test.

## Data Availability

The data that support the findings of this study are available from the corresponding author upon reasonable request.
